# Supplementary material for: Development of a targeted sequencing approach to identify prognostic, predictive and diagnostic markers in paediatric solid tumours
Source: Oncotarget. 2017 Dec 6;8(67):112036–50. doi: 10.18632/oncotarget.23000 (PMC5762377; doi:10.18632/oncotarget.23000)
Supplement: Supplementary file 1 [file oncotarget-08-112036-s001.pdf]

Development of a targeted sequencing approach to identify prognostic, predictive and diagnostic markers in paediatric solid tumours

SUPPLEMENTARY MATERIALS

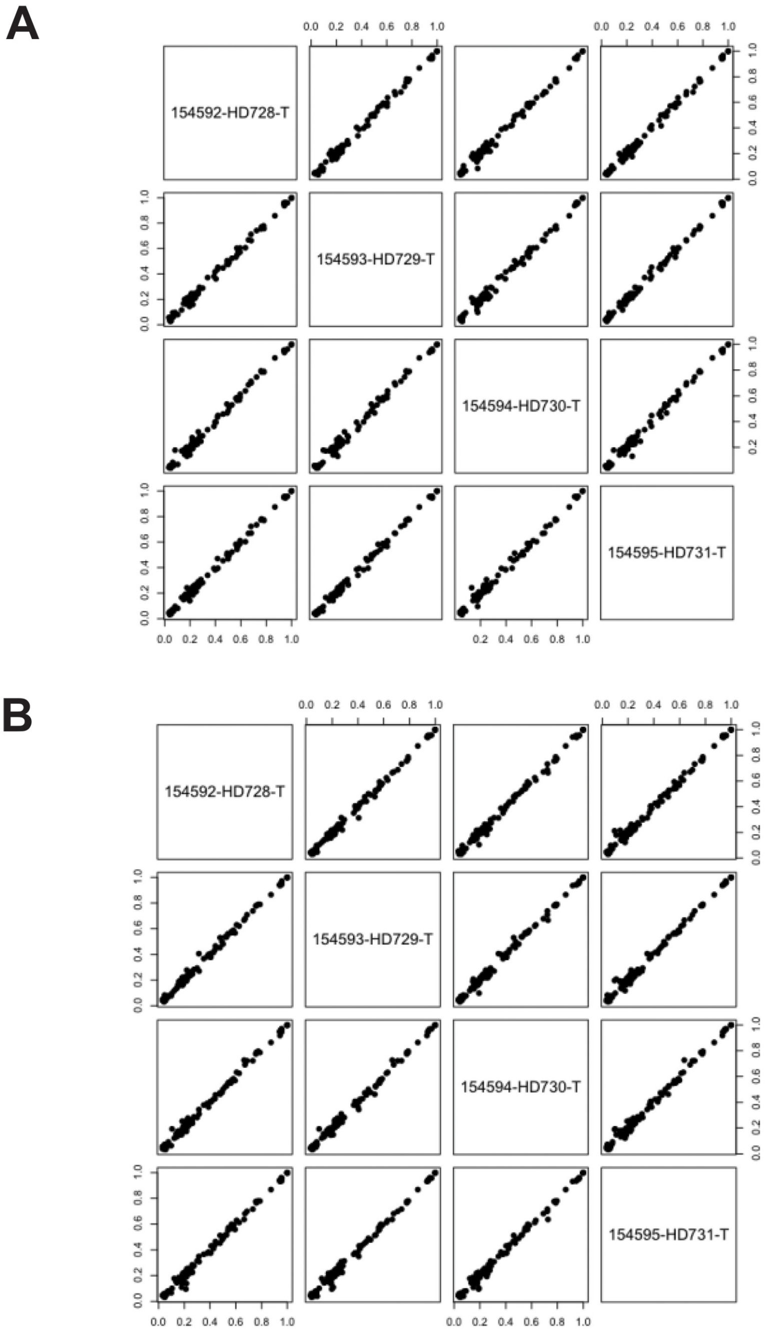

Supplementary Figure 1: Consistency of single nucleotide variants (SNVs) allele frequency in the four HD blends with identical background variants. **(A)** Run_user1, **(B)** Run_user2. In both runs pairwise correlation of 132 SNVs allele frequencies was $r^2 \geq 0.994$ [95%CI:0.9910.996].

A

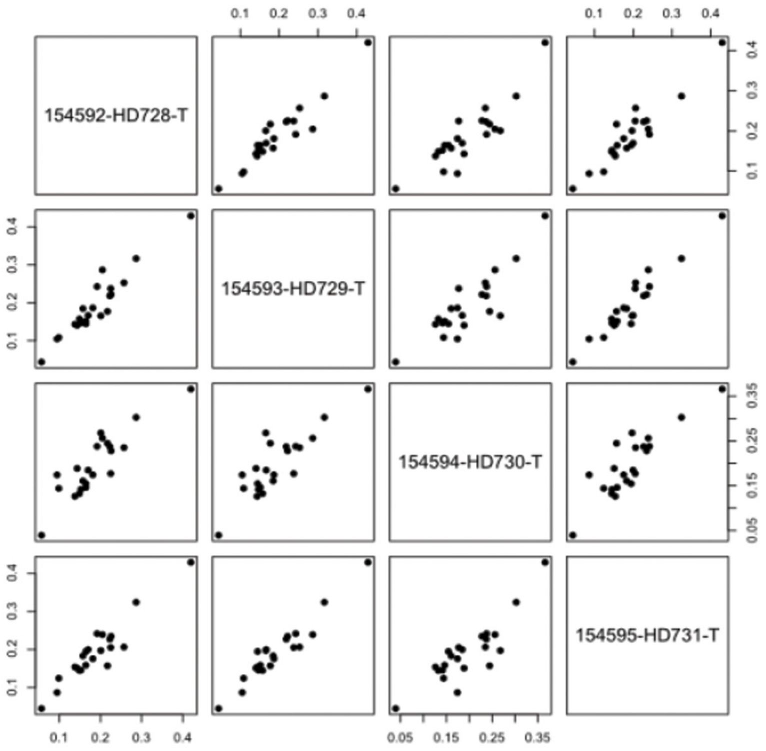

B

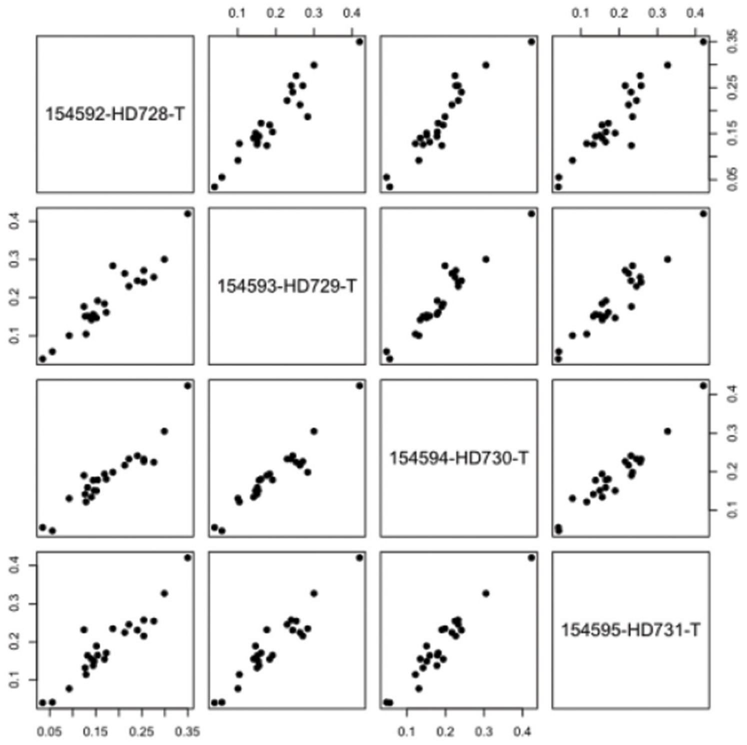

Supplementary Figure 2: Consistency of indels allele frequency in the 4 HD blends with identical background variants. **(A)** Run_user1, **(B)** Run_user2. In both runs pairwise correlation of 22 indels allele frequencies was $r^2 \geq 0.785$ [95%CI:0.652-0.919].

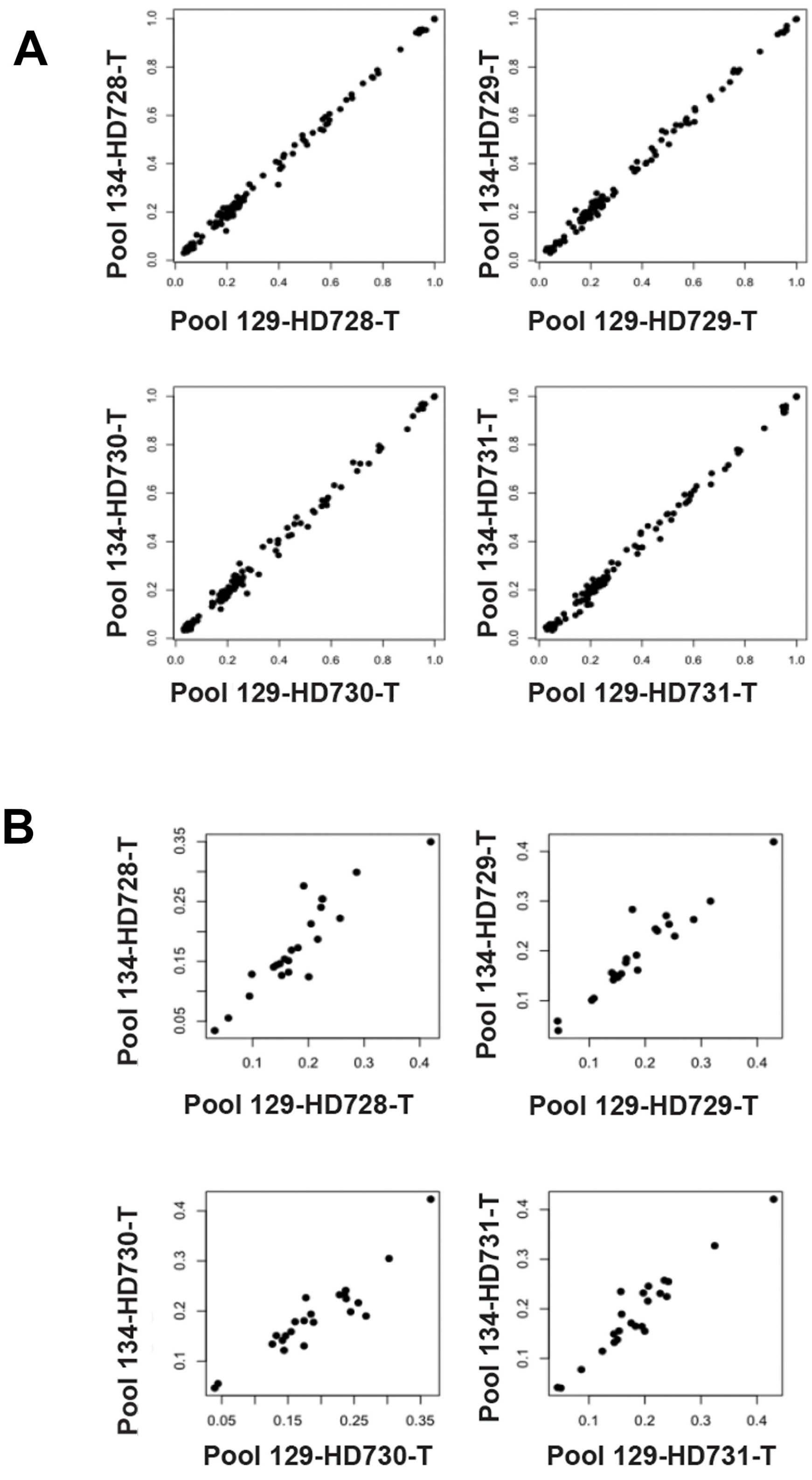

Supplementary Figure 3: Pairwise correlation of variant allele frequency for each of the four HD blends between the two runs (x axis correspond to run_user1 and y axis to run_user2) **(A)** Single nucleotide variants (SNVs) and **(B)** Insertion-deletions (indels). Pair-wise correlation was $r^2 \geq 0.995$ [95%CI:0.993-0.997] for SNVs and $r^2 \geq 0.827$ [95%CI: 0.716-0.937] for indels.

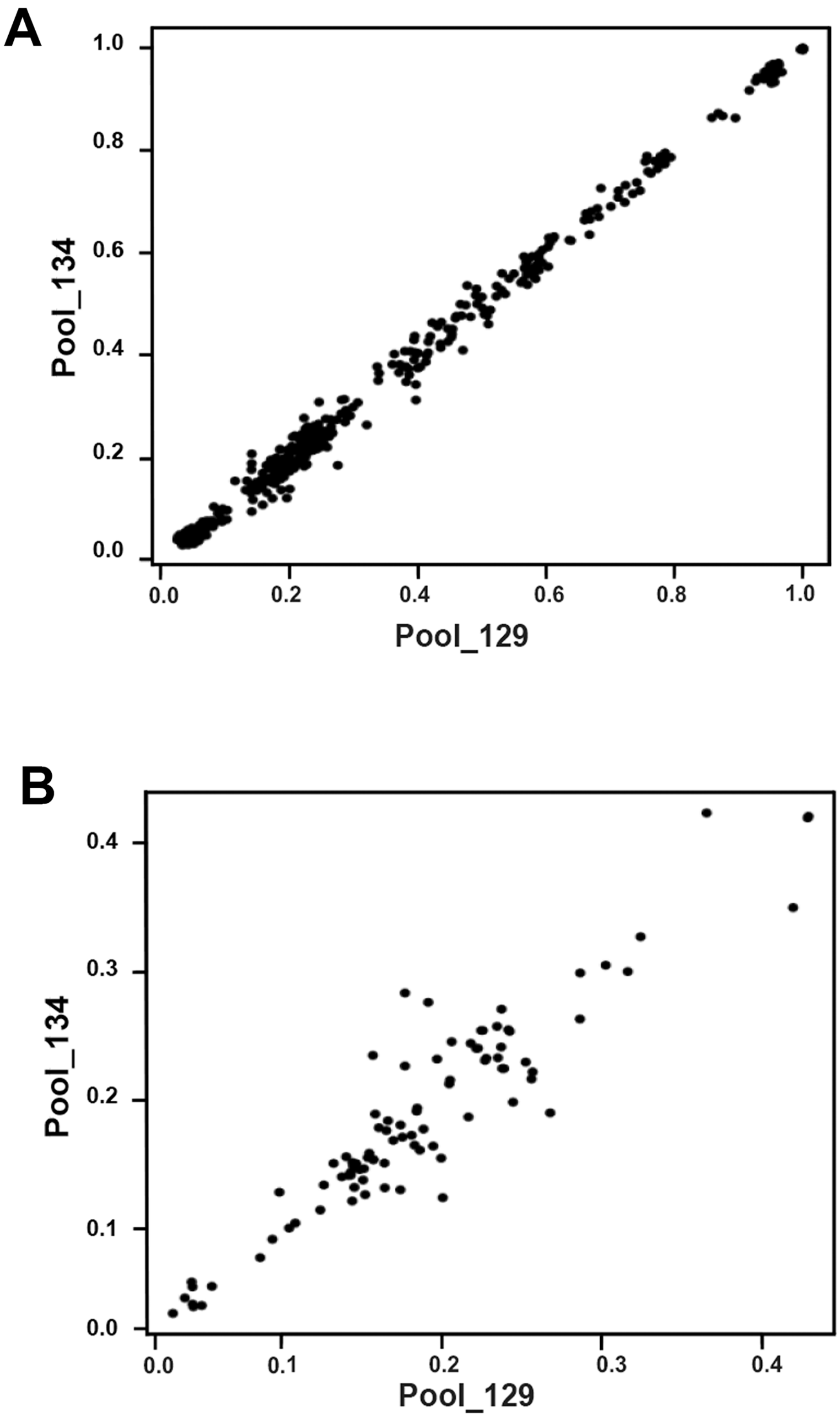

Supplementary Figure 4: Overall correlation of variant allele frequency (VAF) for the HD blends between the two runs analysing the four samples together (x axis correspond to run_user1 and y axis to run_user2) **(A)** Single nucleotide variants (SNVs) and **(B)** Insertion-deletions (indels). Overall correlation was $r^2 \geq 0.996$ [0.995-0.997] for SNVs and $r^2 \geq 0.827$ [95%CI: 0.716-0.937] for indels.

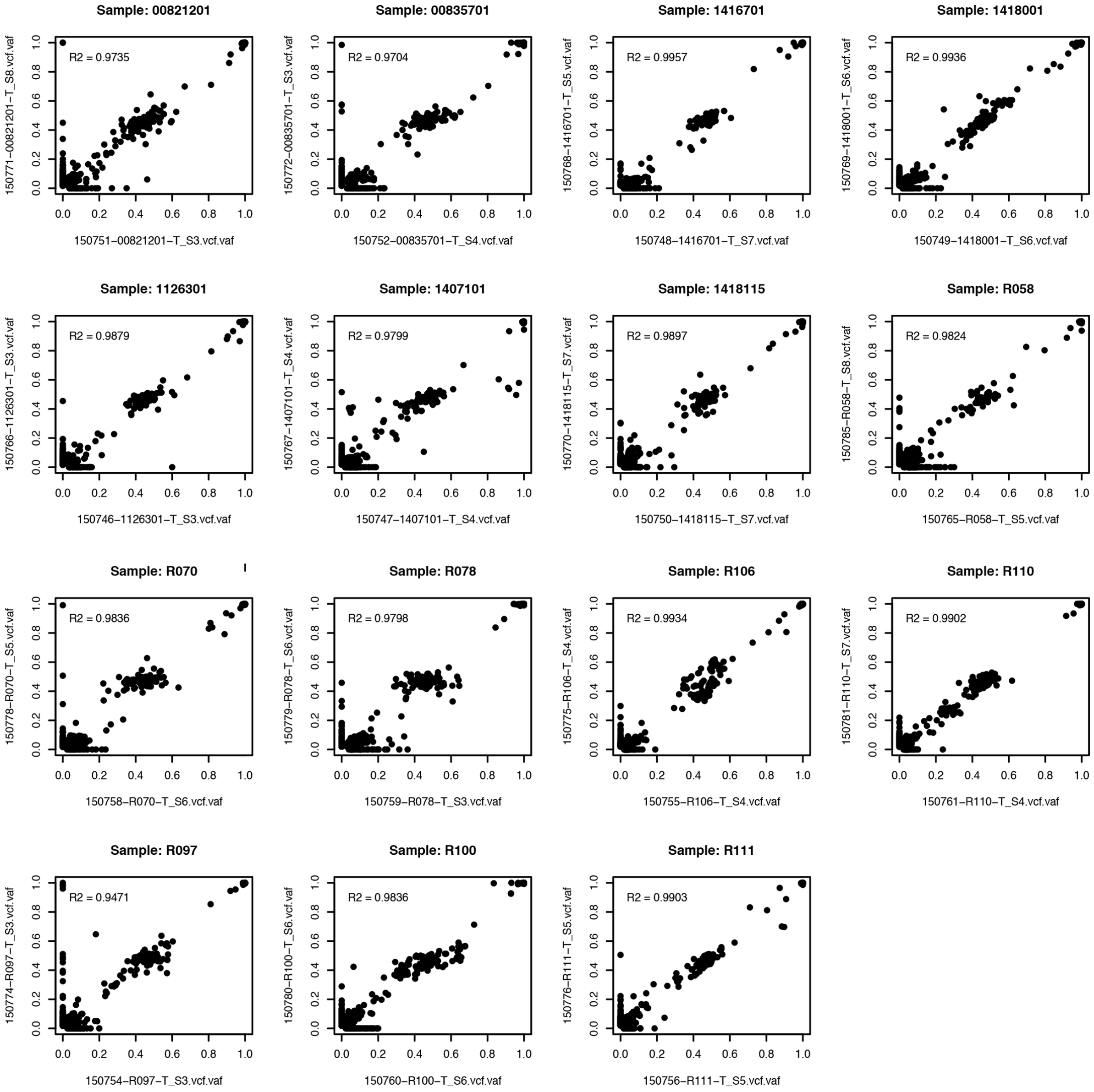

Supplementary Figure 5: Correlation of variant allele frequency found between the 15 formalin-fixed paraffin embedded (FFPE, x axis) and fresh frozen (FF, y axis) paired samples.

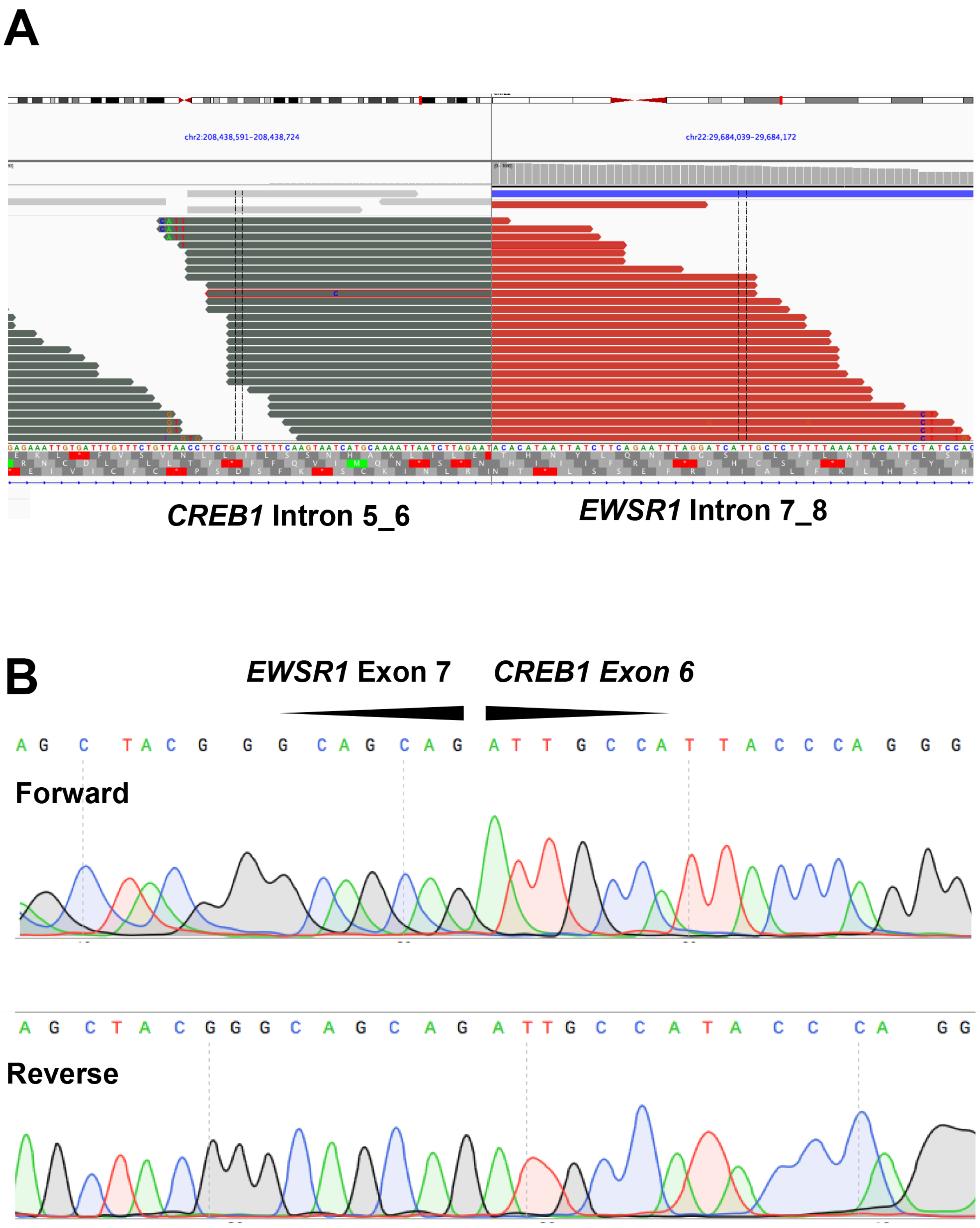

Supplementary Figure 6: *EWSR1-CREB1* translocation in a sarcoma sample known to be positive for this translocation **(A)** Integrative Genomics Viewer plot (IGV) identified by the panel and **(B)** electropherogram by Sanger sequencing.

Supplementary Table 1: (A) List of the 78 genes cover by the paediatric panel. The genes were classified according to whether alterations in that gene have been shown to be predictive biomarker (level 1), prognostic biomarker (level 2), diagnostic biomarker (level 3), potentially targetable biomarkers with inhibitors available or under development (level 4), known germline or high risk single nucleotide polymorphism (level 5) or unclear significance, research only (level 6). The table includes tumour type were alterations have been reported, molecules targeting those genes and clinical trials available. (B) List of the 901 target regions included in the capture panel including exonic and intronic positions selected per gene. ***Please note that intron region chosen for PAX3/7 should have been intron between exons 7 and 8 (PAX3: NM_181457, PAX7: NM_002584) instead of intron between exons 6 and 7***

See Supplementary File 1

Supplementary Table 2: (A) List of background single nucleotide variants (SNVs, n=163) and insertion-deletion (indels n=34) on the four HD cell blends. (B) List of cancer-specific variants at known variant allele frequency (SNVs, n=61; indels n=17) and wild type sites used to determine specificity (n=87) on the four HD cell blends

See Supplementary File 2

Supplementary Table 3: List of all samples used, including: tumour content, cellularity, tumour type and known genetic alterations

See Supplementary File 3

Supplementary Table 4: QC used to determine underperforming regions across the four HD cell blends and four formalin-fixed paraffin embedded samples (FFPE). (A) Mean coverage for each targeted region. (B) Underperforming targeted regions. (C) Percentage of GC content in each targeted region

See Supplementary File 4

Supplementary Table 5: Quality Control metrics for the 132 samples used

See Supplementary File 5

Supplementary Table 6: Coverage metrics for the 132 samples used

See Supplementary File 6

Supplementary Table 7: (A) Variant allele frequency observed by the panel against expected by droplet digital PCR on the HD cell blends for the cancer specific single nucleotide variants and insertion-deletions (SNVs, n=61 and indels n=17) and (B) List of the cancer specific true negative sites (SNVs, n=87 and indels n=3)

See Supplementary File 7

Supplementary Table 8: Variant allele frequency observed on the HD cell blends for the selected single nucleotide variants (SNV, n=132) and insertion deletions (indels, n=27) used for the assessment of precision, sensitivity and specificity

See Supplementary File 8

Supplementary Table 9: Samples with known alterations by other methodologies including 13 cell lines with 30 alterations and 28 clinical samples (14 formalin-fixed paraffin embedded, FFPE and 14 fresh frozen, FF) with 60 alterations

See Supplementary File 9
